# *Spartina alterniflora* Leaf and Soil Eco-Stoichiometry in the Yancheng Coastal Wetland

**DOI:** 10.3390/plants10010013

**Published:** 2020-12-23

**Authors:** Xueyan Zuo, Lijuan Cui, Wei Li, Yinru Lei, Zhiguo Dou, Zhijun Liu, Yang Cai, Xiajie Zhai

**Affiliations:** 1 Institute of Wetland Research, Chinese Academy of Forestry, Beijing Key Laboratory of Wetland Ecological Function and Restoration, Beijing 100091, China; zxyeco@caf.ac.cn (X.Z.); wetlands207@caf.ac.cn (W.L.); leiyinru@caf.ac.cn (Y.L.); douzhiguo@caf.ac.cn (Z.D.); lkylzj@caf.ac.cn (Z.L.); caiy@caf.ac.cn (Y.C.); zhaixiajie@caf.ac.cn (X.Z.); 2 Beijing Hanshiqiao National Wetland Ecosystem Research Station, Beijing 101399, China

**Keywords:** Yancheng Elk Nature Reserve, ecological stoichiometry, leaf-soil, alien invasive plants

## Abstract

Carbon, nitrogen, and phosphorus—nutrient and restrictive elements for plant growth and important components of the plant body—are mainly transferred and exchanged between plants and the soil environment. Changes in the carbon, nitrogen, and phosphorus eco-stoichiometry greatly impact the growth and expansion of *Spartina alterniflora*, and understanding these changes can reveal the nutrient coordination mechanism among ecosystem components. To explore the relationship between leaf and soil eco-stoichiometry and determine the key soil factors that affect leaf eco-stoichiometry, we collected leaf and soil samples of *S. alterniflora* at different tidal levels (i.e., 1, 3, and 5 km away from the coastline) in a coastal wetland in the Yancheng Elk Nature Reserve, Jiangsu province. We measured the leaf and soil carbon, nitrogen, and phosphorus contents and ratios, as well as the soil salinity and soil organic carbon. The results revealed the following. (1) The leaf stoichiometric characteristics and soil properties of *S. alterniflora* differed significantly between tidal levels; for example, total carbon, nitrogen, soil organic carbon were detected at their highest levels at 3 km and lowest levels at 5 km. (2) Significant correlations were detected between the leaf stoichiometric characteristics and soil characteristics. Additionally, nitrogen limitation was evident in the study area, as indicated by the nitrogen–phosphorus ratio being less than 14 and the soil nitrogen–phosphorus ratio being less than 1. (3) Soil salinity and the soil carbon–nitrogen ratio were shown to be the key factors that affect the eco-stoichiometric characteristics of *S. alterniflora*. These findings furthered our understanding of the nutrient distribution mechanisms and invasion strategy of *S. alterniflora* and can thus be used to guide *S. alterniflora* control policies formulated by government management departments in China.

## 1. Introduction

The coastal wetland ecosystem is in the transitional zone between land and ocean and is one of the most diverse and valuable ecosystems in the world. With unique ecological processes, this ecosystem is functional in the maintenance of the coastline and its inhabitants, e.g., preventing coastal erosion, regulating climatic factors, purifying water quality, and providing habitats for organisms [1,2,3,4]. Due to the fragility of this ecosystem, it is vulnerable to the threat of species invasion. *Spartina alterniflora*, a species native to the Atlantic coasts of Canada and the United States, was introduced into China in the 1980s to help increase sediment accumulation and improve erosion resistance [5]. After nearly 40 years, *S. alterniflora* has occupied ~50,000 hectares of coastal wetlands from Hainan Province to Liaoning Province in China [6]. The introduction of *S. alterniflora* has been shown to have numerous beneficial effects, e.g., providing a living space for crabs, effectively protecting the coastline of China, and reducing the impact of storms, and because its rhizomes have a good carbon (C) fixation effect. However, an increasing number of studies have shown that the invasion of *S. alterniflora* has had deleterious effects. For example, the continuous expansion of *S. alterniflora* has seriously threatened the survival of indigenous plants such as *Phragmites australis*, *Suaeda salsa*, and mangrove; has gradually reduced the *Ceratophyllum demersum* community, which has adversely affected the wetland birds that depend on it for survival; and has affected the nutrient cycle of the ecosystem [7,8,9,10]. However, a unified theory has not yet been formed of the relationship between the invasion of *S. alterniflora* and the soil ecosystem [11,12,13].

The invasion of alien plants impacts the environment, economy, society, and functioning of ecosystems and is one of the most important threats facing the global ecosystem [14]. The successful invasion of alien plants involves many factors, including their own biological characteristics and non-biological environmental factors [15,16,17]. In the competition for habitat resources, invasive species often have better resource acquisition strategies than local plants. In resource-rich environments, invasive plants usually have a lower carbon (C) nutrition ratio and higher nitrogen (N)/phosphorus (P) ratios, while in resource-limited environments, invasive plants show higher C assimilation rates, N utilization rates, and energy-utilization efficiency [18,19,20]. Therefore, the successful invasion of alien plants is often related to the conversion and utilization of C, N, and P. Ecological stoichiometry (hereafter “eco-stoichiometry”) mainly refers to the stoichiometric relationship of C, N, and P. Eco-stoichiometric investigations can reveal the theory of chemical transformation of nutrients and the nutrient regulation mechanism among the various components of the ecosystem and can help to increase our understanding of the responses of the plant body structure to nutrient elements and energy balance [21,22]. C, N, and P are important components of plant bodies, as well as the nutrient elements and restrictive elements for plant growth [23,24], which are mainly exchanged between plants and the soil environment [25]. The soil C/N and C, N, and P contents are important indicators of the soil organic matter composition and the stability of how the plant leaf C/N ratio and soil nutrients interact with and restrict each other [26]. Therefore, eco-stoichiometry-based investigations of the coordination mechanism between plant and the soil environment can clarify the nutrient absorption mechanism of invasive species as well as the important factors that drive the dynamic nutrient balance in the environment.

Previous studies on the eco-stoichiometry of *S. alterniflora* have mainly focused on the response of plants to soil nutrients. For example, Xie et al. [27] found that the nutrient content of sediment increased in the early stage of invasion but decreased significantly in the later stage of invasion; however, Liao et al. [28] compared the decomposition of *S. alterniflora* litter and found that the C and N reserves increased. Some studies have shown that *S. alterniflora* invasion reduces soil nutrients [29], but other studies have shown that *S. alterniflora* invasion has little effect on the soil properties and C and N storage [30]. Undoubtedly, a feedback mechanism exists between plants and soil [31]. Although forest plant-soil eco-stoichiometry has been relatively well studied in terrestrial ecosystems [32,33], the relationship between the eco-stoichiometry of wetland invasive plants and the soil environment remain poorly understood. Therefore, we hypothesized that the nutrient utilization of *S. alterniflora* and the corresponding soil stoichiometric relationship between its leaves and the soil environment are important reasons for its successful invasion. This study is of great significance as it furthers our understanding of the nutrient balance and dynamic changes of coastal wetland systems.

In this study, we assessed the relationship between *S. alterniflora* leaf and soil eco-stoichiometry and determined the key soil factors that affect leaf eco-stoichiometry between three different tide levels (i.e., 1, 3, 5 km from the coastline, respectively) in the Yancheng coastal wetland in the Dafeng Elk Nature Reserve. The leaf eco-stoichiometry of *S. alterniflora*, including the total C (TC.L), total N (TN.L), total P (TP.L), C/N ratio (C/N.L), C/P ratio(C/P.L), N/P ratio (N/P.L), corresponding soil stoichiometry, including total C (TC.S), total N (TN.S), total P (TP.S), C/N ratio (C/N.S), C/P ratio (C/P.S), N/P ratio (N/P.S); soil salinity (S.S), and soil organic C (SOC.S), were measured. We explored the C, N, and P utilization strategies of *S. alterniflora* from the coastline, determined the relationships between *S. alterniflora* leaf and soil eco-stoichiometry, and identified the key soil factors that affected the leaf eco-stoichiometry. This study furthers our understanding of the invasion mechanism and nutrient allocation strategy of *S. alterniflora* and provides a theoretical basis for the formulation of control policies for this introduced species.

## 2. Materials and Methods

### 2.1. Study Site

**Figure 1:**
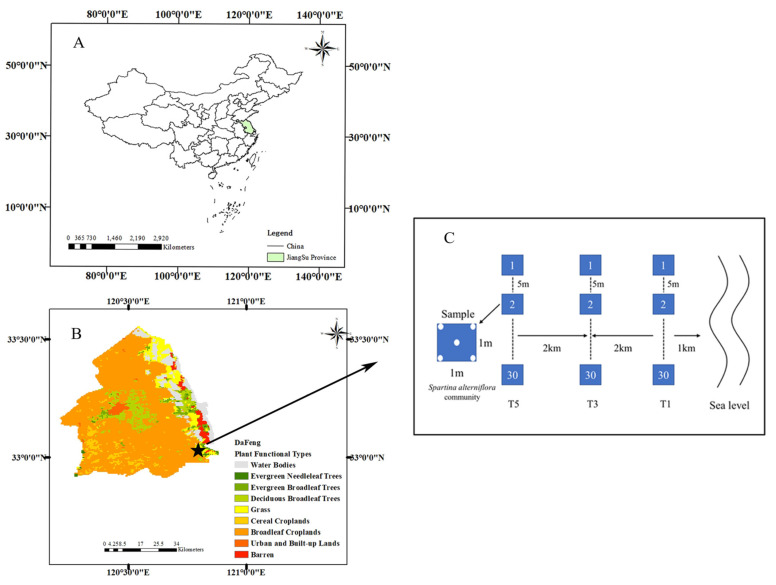
Location of Dafeng Elk Nature Reserve in Jiangsu, China (**A**), the classifications from the MODIS Land Cover Type Product (MCD12Q1) (**B**), and the sampling design used in the present study (**C**). T1, T3, and T5 represent three tidal levels, 1, 3, and 5 km from the coastline, respectively. Samples were collected from 1 m × 1 m plots at 30 points along each sample line.

The Yancheng Dafeng Elk Nature Reserve (Longitude: 120°47′~120°53′ E, Latitude: 32°59′~33°03′ N) is located in the eastern part of Jiangsu province, near to the Yellow Sea, and occurs at the transition zone between a subtropical and a warm temperate zone (Figure 1). Covering an area of 2666 ha, it is the largest elk nature reserve worldwide with the largest number of wild elk populations [34]. In January 2002, the reserve was included in the list of important international wetlands by the United Nations Wetland Conservation Organization as a permanent protected area. The average annual temperature, annual precipitation, and annual frost-free period in the area are 13.5 ℃, 800–1500 mm, and 209–218 days, respectively. In addition, the tidal range is 3.7 m, and the tidal cycle is irregular semi-diurnal [35]. Within the reserve boundary, there are large areas of beaches, swamps, saline-alkali land, and abundant animal and plant resources. Plants mainly include *P. australis*, *S. alterniflora*, and *Imperata cylindrica*, among other species. Among them, *S. alterniflora* was introduced to Yancheng in 1979 [36] and has become the dominant plant with the largest distribution area in the Yancheng coastal wetland.

### 2.2. Sample Collection and Measurement

In August 2019, three sample lines parallel to the coastline were set up in the core area of the Dafeng Elk Nature Reserve in Yancheng at distances of 1, 3, and 5 km from the coastline. Sample lines were separated by intervals of 2 km. *S. alterniflora* plants and soil samples were collected from 30 sample points (1 × 1 m plots) along each sample line, and the interval of sample points was greater than 5 m. Plants showing good growth were randomly selected at each sample point. The leaves in the upper, middle, and lower parts of each plant were collected. Soil samples were collected (depth: 0–20 cm) in each sample plot according to the five-point sampling method and mixed evenly to form a composite soil sample. To avoid leaf nutrient consumption, the samples were quickly transported to the laboratory and dried in an oven (105 °C, 10 min; 80 °C, and 10 h of drying to constant weight). The soil samples were air-dried after removing the impurities of plants and stones, and they were passed through a 100-mesh sieve to determine the content of soil organic carbon, total carbon, total nitrogen, and total phosphorus, and a 20-mesh sieve was used to determine the soil salinity.

The total soil carbon content is the sum of organic carbon and inorganic carbon content, and the carbon in plants is mainly organic carbon, so the total carbon content of plants is the organic carbon content. A vario PYRO cube (Elemental, Langenselbold, Germany) elemental analyzer was used to measure the C and N contents in plants and soil samples, the sample was decomposed by high-temperature combustion, and the mixed gas was automatically determined by the thermal conductivity detection system [37]. The total P content was measured using the molybdenum antimony colorimetric method (Cytation, Biotek, Winooski, VT, USA). Soil organic C was measured using spectroscopy photometer colorimetry (Cytation, Biotek, Winooski, VT, USA); under heating conditions, the organic carbon is oxidized by potassium dichromate-sulfuric acid solution, and the Cr^6+^ in potassium dichromate is reduced to Cr^3+^, and its content is proportional to the organic carbon content. The absorbance was measured at a wavelength of 585 nm to calculate the organic carbon content. C/N refers to the ratio of total carbon to total nitrogen content, C/P refers to the ratio of total carbon to total phosphorus content, and N/P refers to the ratio of total nitrogen to total phosphorus content. Soil salinity was measured using the conductivity method, after extracting the aqueous solution with a water-soil ratio of 5:1, and then measured with a portable conductivity meter (DDS-307A, Shanghai, China).

### 2.3. Data Analysis

A one-way analysis of variance was conducted using SPSS v22.0 to compare the differences in the eco-stoichiometry of *S. alterniflora* leaves and the soil environment, and the other soil physical and chemical properties at different tidal levels. R (Pearson-R) was used to analyze the correlation between the leaf and soil eco-stoichiometry and other indicators. Finally, we conducted redundancy analyses (RDA) to clarify the key soil indicators that affect the eco-stoichiometry of *S. alterniflora* leaves. RDA allows multiple response variables to be regressed on multiple explanatory variables and can explain a set of response variables through a set of explanatory variables. Therefore, RDA was selected for analysis in this study instead of principal component analysis (PCA). All statistical tests were performed at the 0.05 level of significance. Data were visualized mainly using Origin2020 and R.

## 3. Results

### 3.1. Stoichiometric Characteristics of C, N, and P in Leaves of S. alterniflora Leaves

**Figure 2:**
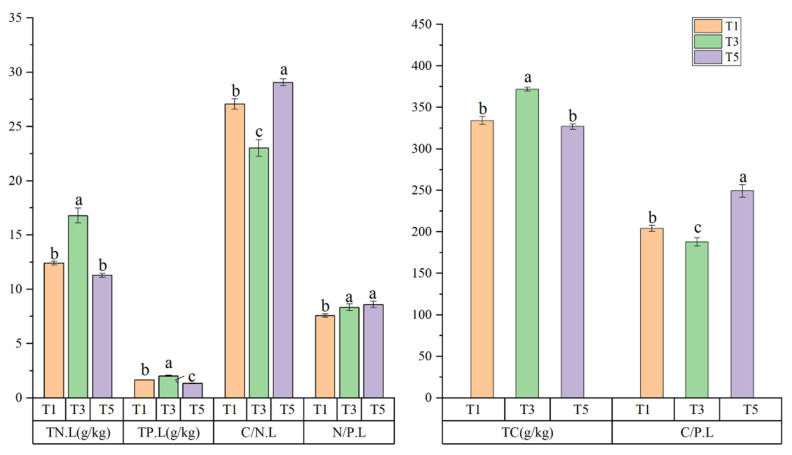
Carbon (C), nitrogen (N), and phosphorus (P) levels and their ratios in *Spartina alterniflora* leaves at different tidal levels: 1, 3, and 5 km from the coastline (T1, T3, and T5, respectively). Lowercase letters represent differences at *p* < 0.05. TC.L: soil total C content; TN.L: leaf total N content; TP.L: leaf total P content; C/N.L: leaf C/N ratio; C/P.L: leaf C/P ratio; N/P.L: leaf N/P ratio.

The stoichiometry of C, N, and P of *S. alterniflora* leaves varied greatly between the different tidal levels (*p* < 0.05) (Figure 2). The TN.L, TP.L, and TC.L contents were the highest at T3 (3 km from the coastline) (16.79 ± 0.69 g/kg, 2.03 ± 0.06 g/kg, 371.90 ± 2.50 g/kg, respectively), intermediate at T1 (12.40 ± 0.18 g/kg, 1.65 ± 0.02 g/kg, 334.18 ± 4.71 g/kg, respectively), and lowest at T5 (11.28 ± 0.16 g/kg, 1.31 ± 0.04 g/kg, 326.93 ± 3.32 g/kg, respectively). All differences were significant (*p* < 0.05), except for the TN.L contents between T1 and T5. The C/N.L and C/P.L levels were the highest at T5 (29.07 ± 0.32, 249.23 ± 7.58, respectively) and lowest at T3 (27.07 ± 0.47, 204.03 ± 3.75, respectively). All differences were significant (*p* < 0.05). The N/P.L content differed from the other indicators in that its maximum value was obtained at T5 (8.59 ± 0.28) and its lowest value was obtained at T1 (7.57 ± 0.15). The N/P.L value did not differ significantly between T3 and T5 (*p* > 0.05). Collectively, these results demonstrated that the C, N, and P stoichiometry of *S. alterniflora* leaves differed significantly between the different tidal levels.

### 3.2. Stoichiometric and Other Characteristics of Soil C, N, and P

**Figure 3:**
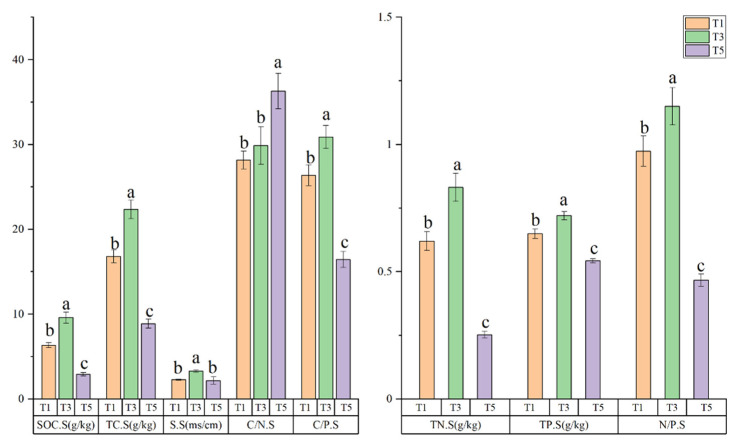
Soil eco-stoichiometry, organic carbon (C), and salinity characteristics at different tidal levels: 1, 3, and 5 km from the coastline (T1, T3, and T5, respectively). Lowercase letters represent differences at *p* < 0.05. SOC.S: soil organic C content; TC.S: total soil C content; TN.S: soil total nitrogen (N) content; TP.S: soil total phosphorus (P) content; S.S: soil salinity; C/N.S: soil C/N ratio, C means TC.S; C/P.S: soil C/P ratio, C means TC.S; N/P.S: soil N/P ratio.

Significant differences were detected in the soil C, N, and P stoichiometry, soil organic C content, and salinity at different tidal levels (*p* < 0.05) (Figure 3). The SOC.S, TC.S, TN.S, TP.S, and S.S were the highest at T3 (9.59 ± 31 g/kg, 22.34 ± 1.10 g/kg, 0.83 ± 0.06 g/kg, 0.72 ± 0.02 g/kg, 3.30 ± 0.13 ms/cm respectively), intermediate at T1, and lowest at T5 (2.91 ± 0.21 g/kg, 8.88 ± 0.51 g/kg, 0.25 ± 0.01 g/kg, 2.17 ± 0.42, respectively). All differences were significant (*p* < 0.05), except the S.S, which did not differ significantly between T1 and T5. The C/P.S and N/P.S showed the same trend as the other soil indexes, where the highest values were obtained at T3 (30.89 ± 1.37, 1.15 ± 0.07, respectively), followed by T1 and T5. The highest C/N.S value was obtained at T5 (36.29 ± 2.08), but no significant differences were obtained between T1 and T3 (*p* > 0.05). Collectively, these results demonstrated that the C, N, and P stoichiometry of soil, the soil organic C content, and the salinity of the soil differed significantly between the different tidal levels.

### 3.3. Relationship between the Leaf and Soil C, N, P Stoichiometric Characteristics and Other Physical and Chemical Properties

**Figure 4:**
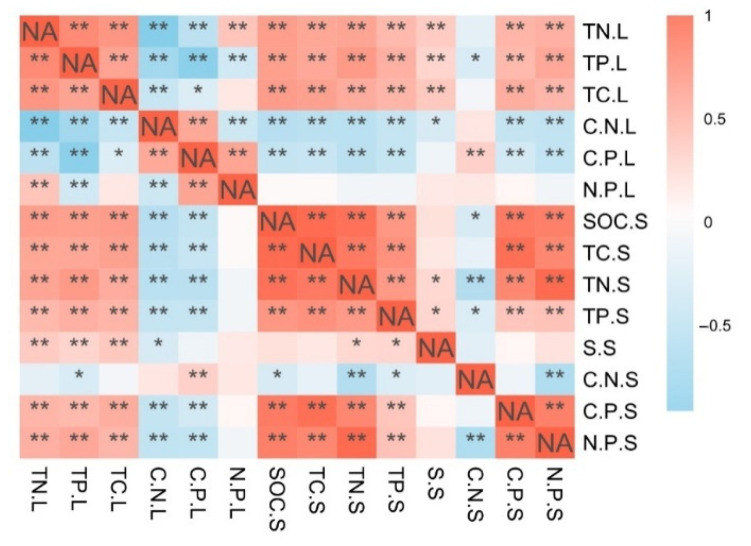
Correlation coefficients between leaf carbon (C), nitrogen (N), and phosphorous (P) stoichiometric characteristics and soil characteristics (C, N, P stoichiometry, organic C, salinity). TC.L: total soil C content; TN.L: total N content of leaves; TP.L: total P content of leaves; C/N.L: leaf C/N ratio; C/P.L: leaf C/P ratio; N/P.L: leaf N/P ratio; SOC.S: soil organic C content; TC.S: soil total C content; TN.S: soil total N content; TP.S: soil total P content; S.S: soil salinity; C/N.S: soil C/N ratio; C/P.S: soil C/P ratio; N/P.S: soil N/P ratio; * indicates *p* < 0.05, ** indicates *p* < 0.01.

The variation in TN, TP, TC, and C:N:P ratios of *S. alterniflora* were related to TC, TN, TP, and S.S content and C/P, N/P in soils but less related to the C/N.S (Figure 4), and N/P.L was less related to the indicators of soils. There was a positive correlation between TC.L, TN.L, TP.L, and soil indicators (TC.S, TN.S, TP.S, SOC.S, S.S, C/P.S, and N/P.S) and a positive correlation between C.P.L and C.N.S. The C/N.L and C/P.L showed a significant negative correlation with SOC.S, TC.S, TP.S, TN.S, S.S, C/P.S, and N/P.S. In addition, the C/N.L showed a negative correlation with S.S (Figure 4). Therefore, the TC, TN, and TP content of *S. alterniflora* leaves increased, but C/N.L and N/P.L ratios decreased with the increasing SOC, TC, TN, TP, C/P, and N/P in the soil.

### 3.4. Effects of Soil Eco-Stoichiometry on Leaf Eco-Stoichiometry

**Figure 5:**
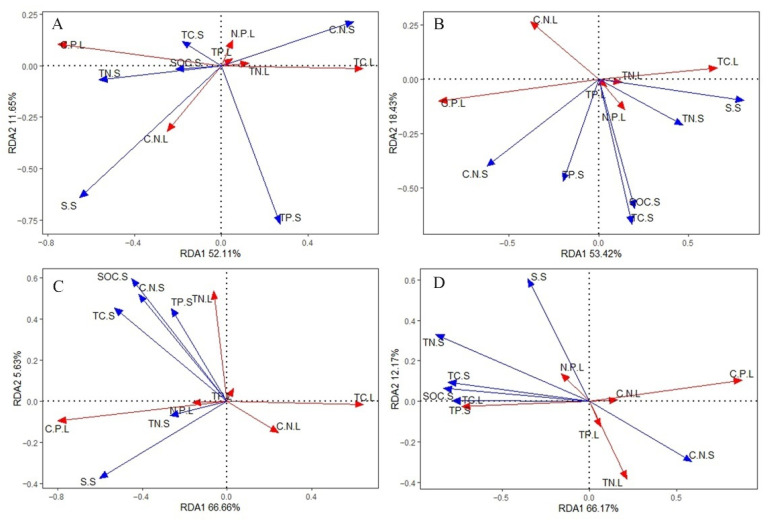
Soil eco-stoichiometry and the relationships between organic carbon (**C**), salinity, and leaf eco-stoichiometry at different tidal levels. Blue and red arrows indicate the soil index and the plant index, respectively. Subfigures (**A**–**C**) represent the sample lines at the three tidal levels: 1, 3, and 5 km from the coastline (T1, T3, and T5, respectively); (**D**) represents all sample points from three tidal levels. TC.L: soil total C content; TN.L: leaf total nitrogen (N) content; TP.L: leaf total phosphorus (P) content; C/N.L: leaf C/N ratio; C/P.L: leaf C/P ratio; N/P.L: leaf N/P ratio; SOC.S: soil organic C content; TC.S: soil total C content; TN.S: soil total N content; TP.S: total soil P content; S.S: soil salinity; C/N.S: soil C/N ratio; C/P.S: soil C/P ratio; N/P.S: soil N/P ratio.

Redundancy analysis (RDA) was used to determine the key factors in soil stoichiometry and other properties that affect the eco-stoichiometry of *S. alterniflora* leaves. In the analyses, the *S. alterniflora* leaf eco-stoichiometry represented the response variable, and the soil indicators represented the explanatory variables. The results of the RDA analysis (Figure 5) revealed that most of the variances in Figure 5A–D were represented by axis 1 (A–D: 52.11%, 53.42%, 66.66%, 66.17%, respectively), and the cumulative interpretation rate of axis 2 reached more than 60% (A–D: 63.76%, 71.85%, 72.2%, 78.34%, respectively). The cosine value of the arrow angle approximates the correlation between the variables: the angle closer to 90° means less correlation among variables, and the angle farther from 90° and closer to 0° or 180° means strong positive or negative correlation. These results showed that S.S, C/N.S, and TP.S had a greater impact on leaf C, N, and P stoichiometry (Figure 5A). At T3, the S.S, C/N.S, SOC.S, and TC.S had a greater impact (Figure 5B), and at T5, the S.S, C/N.S, and TC.S had a greater impact on the leaf stoichiometric characteristics (Figure 5C). Comprehensive RDA analysis results of the three tidal levels revealed that the S.S, C/N.S, SOC.S, TP.S, and TC.S had a greater overall impact (Figure 5D). Collectively, the results demonstrated that S.S and C/N.S greatly impacted the *S. alterniflora* leaf eco-stoichiometric characteristics.

## 4. Discussion

### 4.1. Relationships between the Eco-Stoichiometry of S. alterniflora Leaves and Soil Physical and Chemical Properties

This study demonstrated that *S. alterniflora* leaves and soil C, N, and P stoichiometric characteristics were greatly affected by the tidal level and differed greatly between the different distances from the coastline. Such differences reflect the adaptability of *S. alterniflora* to different environments. C is an important element in the formation of plant bodies. In this study, the C content, C/N ratio, and C/P ratio were observed to differ significantly among the different tide levels, which reflects the different nutrient utilization efficiency of *S. alterniflora* at different distances from the coastline [38]. The N/P ratio in leaves is an important indicator to reflect if plants are restricted by nutrition during their growth [39,40]. The average N/P ratio in this study was 8.17 ± 0.15, which is significantly lower than the average value of other terrestrial and aquatic plants [41,42,43]. Moreover, Jack et al. [39] demonstrated that when the N/P ratio of wetland plant leaves was less than 14, it indicated that the soil N resources were limited. In addition, the N/P ratio in the soil was 0.86 ± 0.04, which is significantly lower than that of leaves. This result indicates that *S. alterniflora* is able to more easily accumulate N to maintain survival and ensure growth when N resources are limited in the soil [44], thus reflecting the growth strategy of *S. alterniflora*. The soil C/P ratio is an important indicator of soil P availability [45,46]. In the present study, we found that the soil C/P ratio was significantly related to leaf C, N, and P contents. Therefore, the availability of soil P is believed to be closely related to leaf nutrient levels.

The transfer of C, N, and P between plants and the soil environment forms part of the C, N, and P cycles within ecosystems. The stability of C, N, and P in the plant leaves affect the soil nutrients and, accordingly, the stability of these nutrient elements in the soil also affect the nutrient availability for plants [47]. The findings of the present study were consistent with this notion, i.e., the C, N, and P contents in the *S. alterniflora* leaves were significantly positively correlated with the soil C, N, and P contents and with the soil organic C content.

### 4.2. Key Soil Factors That Affect Leaf Eco-Stoichiometry

The C, N, and P contents of plants are affected by the comprehensive effects of environmental factors, such as the soil physical and chemical properties and the atmospheric carbon dioxide concentration [48]. In this study, RDA analyses were used to evaluate the key environmental factors that affect the stoichiometric characteristics of leaf C, N, and P. Soil is the main source of plant nutrients [49,50]; therefore, the soil C, N, and P concentrations are expected to determine the concentrations of plant C, N, and P. In addition, our study revealed that soil salinity and soil C/N ratio greatly impacted the eco-stoichiometric characteristics of *S. alterniflora* leaves. We speculated that the C/N ratio is a sensitive indicator of soil quality and has important impacts on the soil C and N cycles and plant growth. Some studies have shown that a low C/N ratio can promote microbial decomposition and N mineralization, while a high C/N ratio can slow down the mineralization of organic matter and organic N and promote the fixation of organic C [51]. In addition, high salinity levels can also slow down the mineralization of organic C [48,52]. A significant correlation was detected between the soil organic C and the leaf C, N, and P levels (*p* < 0.01, Figure 4). Therefore, we showed that the salinity levels and the C/N ratio greatly influenced the leaf stoichiometry, which is similar to the findings of Zhao et al. and Rath et al. [52,53]. In addition, the soil total N, total C, and total P were also shown to affect the leaf stoichiometry, which further confirms the interactions and restrictions between plants and the soil environment.

### 4.3. Limitations of This Study

According to the Koerselman critical threshold theory, we conclude that *S. alterniflora* in this period is mainly restricted by N [43], but due to the influence of climate characteristics, species types, and the invasion period and growth period of *S. alterniflora*, this result has certain limitations. Research has shown that *S. alterniflora* is mainly restricted by N in the initial stage; along with its growth, the nitrogen fixation effect of *S. alterniflora* will reduce its dependence on nitrogen, its internal stability will change, and P may become a new limiting factor. In addition to its own nutrient utilization strategy, community environment leaves such as companion species also play an important role [54]. In addition, due to their different statuses in the community during different invasion periods, nutrient utilization and its internal stability will be affected by species’ dominance and ecosystem stability [55]. In the study of Wang, the soil TC, TN, and TP contents were similar to those in this study, but the C/N was lower than in this study. There may have been two reasons for this: one is that his result is a combination of the results of multiple different regions, thus weakening those of the individual regions; another reason is that the plants in this study have a lower decomposition rate due to periodic flooding, resulting in a higher carbon–nitrogen ratio [30]. Therefore, these results need further experiments to confirm and explore the nutrient limiting characteristics and more environmental factors of *S. alterniflora* in different growth stages, invasion periods, and habitats.

## 5. Conclusions

In this study, the distance from the coastline was used as a variable to explore the relationship between the eco-stoichiometric characteristics of *S. alterniflora* leaves and soil salinity, soil organic C, and soil eco-stoichiometry characteristics. In addition, we explored the key factors that affect the stoichiometric characteristics of *S. alterniflora* leaves. The stoichiometric characteristics of *S. alterniflora* leaves and the stoichiometric characteristics and properties of the soil were found to differ significantly between the different tidal levels, and many of the factors were detected at their highest levels at 3 km and at their lowest levels at 5 km, e.g., the leaf total carbon, total nitrogen, and total phosphorus; the soil total carbon, total nitrogen, and total phosphorus; the soil salinity; and the soil organic carbon. Significant correlations were confirmed between the leaf stoichiometry and the soil properties. The N/P ratio of plants was less than 14 and the N/P ratio of soil was less than 1, which was indicative of N limitation in the study area. Soil salinity and the soil C/N ratio were revealed to be the key factors that affect the stoichiometric characteristics of C, N, and P of *S. alterniflora* at different distances from the coastline. Considering variable distances from the coastline distance, soil salinity and soil C/N ratio were the key factors that affected the stoichiometric characteristics of *S. alterniflora*. Therefore, soil salinity and C/N ratio are important factors to be considered when regulating the growth of *S. alterniflora*. These results only represent the preformation of this growth stage. The next step will be to conduct experiments to confirm and explore the nutrient limitation characteristics and main factors of *S. alterniflora* eco-stoichiometric in different growth stages and different invasion periods. This conclusion is of great significance for both the management of coastal wetlands and to ensure the health of the ecological functions of the ecosystem at this stage.
